# 

**Published:** 1881-08

**Authors:** 


*Vol. XXXV.* **AUGUST, 1881.** *No. 8.*

THE

### Dental Register,

A MONTHLY JOURNAL,

DEVOTED TO THE INTERESTS OF THE PROFESSION.

EDITED BY J. TAFT, D. D. S.

PUBLISHED BY

SPENCER & CROCKER,

OHIO DENTAL DEPOT,

NOS. 117, 119, 121 WEST FIFTH STREET, CINCINNATI, OHIO.

*TERMS—$2.50 Per Annum, in Advance. Single Copies, 25 Cents.*

